# The Enduring Burden of Advanced Human Immunodeficiency Virus Disease

**DOI:** 10.1093/cid/ciaa265

**Published:** 2020-05-05

**Authors:** Nathan Ford, Eric Goemaere, Katherine Hildebrand, Carmen Perez-Casas

**Affiliations:** 1 Human Immunodeficiency Virus, Hepatitis, and Sexually Transmitted Infections Department, World Health Organization, Geneva, Switzerland; 2 Centre for Infectious Disease Epidemiology and Research, School of Public Health and Family Medicine, Faculty of Health Sciences, University of Cape Town, Cape Town, South Africa; 3 Southern Africa Medical Unit, Médecins Sans Frontières, Cape Town, South Africa; 4 Unitaid, Geneva, Switzerland

**(See the Brief report by Nasuuna et al on pages 2497–99.)**

To the Editor—Over the past 20 years the role of CD4 cell counts in human immunodeficiency virus (HIV) care has evolved. Initially used as a clinical tool to assess disease severity and urgency of care, CD4 cell counts became the main way to assess eligibility for starting antiretroviral therapy (ART) and assess efficacy and adherence to treatment. Then from 2016 onward, most HIV guidelines worldwide shifted to recommending starting ART irrespective of CD4 cell count, with viral load recommended as the preferred way to assess treatment efficacy and program performance; funding was directed away from supporting CD4 cell count capacity to increase capacity to measure viral load [1, 2].

Nevertheless, CD4 remains a key diagnostic tool to direct the diagnosis of specific life-threatening opportunistic infections. Although HIV-associated mortality has declined as access to treatment has increased—globally, 24.5 million of the 37.9 million people living with HIV are receiving ART—this decline in mortality has been far slower than anticipated [3]. In 2019, 770 000 people died of HIV-related causes [4]. Several recent studies have shown that although, on average, CD4 status at start of ART has increased, the proportion of patients starting or restarting treatment with advanced HIV disease (ie, a CD4 cell count <200 cell/mm^3^) remains high [5, 6].

A study published in this issue of *Clinical Infectious Diseases* of over 40 000 HIV-positive adults starting ART in Uganda found that in 2018 a quarter (24%) of people tested had advanced HIV disease [7]. In this study, the proportion of people presenting with advanced HIV disease declined only modestly during the study period, from 29% to 24% between 2013 and 2018; over the same 6-year period, the proportion of people presenting with very advanced HIV disease (a CD4 cell count <100 cells/mm^3^) only decreased from 14% to 12% [7].

Guidelines issued by the World Health Organization (WHO) in 2017 recommend a package of screening, prophylaxis, and treatment of opportunistic infections, rapid ART initiation, and adherence support for patients with advanced HIV disease [8]. This core package targets 2 of the leading causes of hospitalization and death of people with HIV—TB and cryptococcal meningitis—and is derived from 2 randomized trials that showed a 27% and 28% mortality reduction when delivering components of the advanced HIV disease package [9, 10]. The advanced HIV disease package, recommended by a growing number of national HIV guidelines and supported by major donors, was designed as a simple approach to rapidly diagnose, prevent, and manage leading causes of death. The package is intended as part of a public health approach that can be delivered by lesser trained health workers in primary care settings, to be adjusted to maximize potential at primary healthcare level.

Médecins Sans Frontières is working with ministries of health across several high-HIV burden countries in southern Africa to assess the feasibility of delivering the advanced HIV disease package in rural, remote primary care settings [11]. Nurses, and in certain contexts lay health workers, conduct point of care testing for CD4 cell count for patients presenting or re-presenting to care; those with a CD4 <200 cell count <200 mm^3^ receive cryptococcal antigen (CrAg) testing and TB LAM, and those who test positive are started on treatment where feasible or referred to hospital. In these settings, around a quarter of patients with advanced HIV disease test positive for TB lateral flow urine lipoarabinomannan assay (TB-LAM), whereas around 5–10% test positive for CrAg. This represents a considerable burden of life-threatening disease that can be detected and treated through administration of a simple package of diagnostics.

With a focus on these rapid diagnostics tools and priority medicines within the WHO recommended package of care, the Unitaid-supported advanced HIV disease initiative (implemented with The Clinton Health Access Initiative in collaboration with key partners), is leading a market-shift to enable prompt access, wider use, and supply reliability of optimal products needed to combat the persistent mortality rates [12]. Point-of-care diagnostic tools are lacking for other leading causes of death such as severe bacterial infections and toxoplasmosis, and WHO will give consideration to revising the package of care for advanced HIV disease if such tools are developed.

Clearly, the gateway for responding to advanced HIV disease is CD4 cell count testing. In the study from Uganda, and despite the slow decrease in the burden of advanced HIV disease, the proportion of individuals receiving a baseline CD4 declined from 73% in 2013 to 21% in at the end of 2018. Considering the poor diagnostic accuracy of clinical staging alone—in this study, among those patients with advanced HIV disease, 83% had WHO Stage I or II—a considerable number of patients with advanced HIV disease would have been missed [7, 13]. Simple semi-quantitative point-of-care, maintenance free, low-cost rapid tests for CD4 cell count are becoming available that can support increased access to appropriate and timely identification of those in advanced stages of the disease.

It is important to note that this study was limited to patients newly initiating ART, and this provides only a partial picture of the overall burden of advanced HIV disease. A study from South Africa found that 57% of patients with a very low CD4 cell count (<50 cells/mm^3^) were treatment-experienced [14]. Advanced HIV disease is not simply, or even mainly, a problem of late diagnosis and presentation to care: a growing proportion of patients with advanced HIV disease have previously engaged in care, started ART, subsequently disengaged from care, and represent to care with advanced HIV disease—if indeed they represent at all. Several studies have shown that negative provider attitudes are cited as reasons for disengagement and nonreturn to care, and in South Africa and elsewhere, “Welcome Back” services have been established to encourage people to reengage with care in a nonjudgemental and friendly environment [15, 16].

This study nevertheless adds to the growing body of evidence that responding to advanced HIV disease continue to be an important current challenge for the global HIV response. Future reporting of the distribution of both adults and children with low CD4 cell counts is encouraged to support programme planning and procurement of essential, life-saving diagnostics and therapeutics to reduce HIV-associated mortality.

## Note

***Potential conflicts of interest.*** The authors: No reported conflicts of interest. All authors have submitted the ICMJE Form for Disclosure of Potential Conflicts of Interest.
